# Socio-cultural factors influencing adolescent pregnancy in Ghana: a scoping review

**DOI:** 10.1186/s12884-022-05172-2

**Published:** 2022-11-11

**Authors:** Mustapha Amoadu, Edward Wilson Ansah, Patricia Assopiah, Philomina Acquah, Joyce Evelyn Ansah, Eunice Berchie, Doris Hagan, Elsie Amoah

**Affiliations:** Department of Health, Physical Education and Recreation, University of Cape Coast, Cape Coast, Ghana

**Keywords:** Socio-cultural factors, Adolescent pregnancy, Ghana

## Abstract

**Background:**

Adolescent pregnancy is a public health challenge that has well-defined causes, associated health risks, and social and economic consequences for adolescent, their families, communities, and society. The purpose of this scoping review is to summarize studies published on socio-cultural determinants of adolescent pregnancy in Ghana.

**Methods:**

Search for records was done in four major databases, including PubMed CENTRAL, Science Direct and JSTOR. Records from Google and Google Scholar were also added, and results and findings from published and unpublished studies were included. All the 22 studies that met the eligibility criteria, were critically appraised. The guidelines for conducting scoping reviews by Arksey and O’Malley were followed.

**Results:**

The result revealed that poverty, peer influence, low level of education, dysfunctional family, lack of communication between parents and their daughters, lack of sexual and reproductive health education, child marriage, coerced sex, misconception and non-usage of contraceptives, and decline in cultural values such as puberty rites and virginity inspection are some of the determinants of adolescent pregnancy in Ghana. The study also showed that there is a lack of high-quality observational studies that adjust for confounding variables.

**Conclusion:**

Interventions and policies should be designed to take into consideration the needs, context, and background of adolescents. Programmes to enhance adolescent reproductive health need to consider multilevel factors such as person, family, community, institutions, national, and global issues that affect such programmes.

**Supplementary Information:**

The online version contains supplementary material available at 10.1186/s12884-022-05172-2.

## Background

Adolescent pregnancy and its challenges have long been a public health issue globally. Adolescents are people aged 10 to 19 years old [1]. Adolescence is a critical stage in human development because adolescent body undergoes rapid physiological, psychological, and social changes [2]. Also, adolescence is usually regarded as a period of good health and hence adolescents are generally regarded as healthy individuals [2]. However, adolescents are vulnerable and exposed to a variety of health risks, causing a large number of them to die during this stage, and the causes of their deaths are, for the most part, preventable [1].

Adolescent pregnancy is a public health issue with well-defined health risks, social and economic consequences to the individual, their families, communities, and society. In developing countries, an estimated 21 million adolescent girls (15–19 years) become pregnant and about 12 million of these girls give birth each year [3]. Moreover, Neal et al. [4] reported that 2.5 million adolescent girls below the age of 16 give birth yearly. In Ghana, two out of 10 girls become pregnant or welcome their first child before they reach the age of 18 years [5, 6].

Adolescent girls may want to avoid pregnancy; however, they are unable to do so due to misconceptions some adolescents may have about contraception and knowledge gap, where to get contraceptives, and how to use it well [6, 7]. Also, sexuality is still largely a taboo subject in Ghana, and sex education in schools is generally restricted to abstinence messages [6, 8–11], besides, the issue of child marriage is still prevalent in Ghana [6]. Perhaps, these misconceptions, knowledge gaps, child marriage, and lack of sexuality education may be some of the reasons why about 70% of adolescent girls in Ghana have unmet needs for contraception [6].

Adolescent mothers are at risk of pregnancy-related complications such as hypertensive pregnancy disorders, urinary tract infections (UTIs), unsafe abortions and premature rupture of the fetal membrane [12, 13], and STIs, poor nutrition, anaemia, and cesarean delivery [1]. Also, adolescent mothers who deliver through cesarean sections have a higher risk of abnormalities such as placenta previa, placenta abruption, uterine rupture, and placenta accreta in subsequent pregnancies [14]. In addition, babies born to adolescents are more likely to be preterm, have low birth weight, and other severe neonatal conditions [15]. Consequently, adolescent birth complications are the major cause of mortality among girls between 15 and 19 years [1, 6] Moreover, adolescents who are not married and are pregnant may face social consequences such as stigma and rejection from their parents, peers. and community members [8]. Perhaps, in resource constraint communities or nations like Ghana, adolescent pregnancy may jeopardize adolescent education and employment opportunities. Unfortunately, girls (below the age of 18 years) who get pregnant have higher chances of facing violence in a marriage or partnership [1, 6].

Adolescent pregnancy has a distressing economic and psychosocial impact, as well as a negative health impact, that needs urgent attention especially in developing nations [16]. Thus, adolescent pregnancy can cause drastic changes in the lives of adolescent girls, and in some cases, these girls may drop out or have their education interrupted. Moreover, evidence suggests that when compared with pregnant adults (over the age of 19), pregnant young girls are more likely to experience mental health challenges such as stress, depression, hopelessness, despair, low self-esteem, suicidal ideation and attempts, feelings of failure, and, in extreme cases, commit suicide [17]. In addition, adolescent pregnancy stereotypes, stigma, rejection, and isolation may impede their access to healthcare during pregnancy. Perhaps, these negative behaviours (stigma and stereotype) from society and healthcare professionals may increase the likelihood of adolescents avoiding essential maternal care services which increases the propensity of adolescent pregnancy complications.

One of the ways to eliminate or reduce the adverse pregnancy outcomes faced by adolescents is to prevent these young girls from getting pregnant [6–8]. Preventing adolescent pregnancies means understanding the determinants and using effective policies and interventions to enable adolescent make healthier sexual choices [9, 10]. Determinants of adolescent pregnancy such as socio-cultural factors may differ depending on geographical location, and thus, a thorough understanding of these factors is required for practice and policy. The literature on sociocultural factors has a variety of applications in social work, public health, and public policy practice and advocacy. Working on adolescent pregnancy in Ghana may necessitate an assessment of adolescents’ values regarding attitudes and perceptions toward sex, pregnancy and childbirth, and parenthood in the context of family, community, and the larger society. Literature evidence on the socio-cultural determinants of adolescent pregnancy may be critical in informing decisions and practice in social work, public health promotion, and, public health policy. However, despite decades of research on the determinants of adolescent pregnancy there is a lack of review study that has mapped evidence on the sociocultural determinants of adolescent pregnancy in Ghana. Therefore, this study will fill this gap by mapping the evidence on socio-cultural determinants of adolescent pregnancy in Ghana to help inform practice, policy, future studies, and possible systematic reviews. The purpose of this scoping review is to map and summarize studies published on socio-cultural determinants of adolescent pregnancy in Ghana, to help identify research gaps, social and cultural factors and make recommendations to strengthen practice, public health policy formulation and future studies.

## Methods

This scoping review is conducted using the guidelines of Arksey and O’Malley [18]. In addition, the preferred reporting items for systematic reviews and meta-analyses extension for scoping reviews (PRISMA-ScR) checklist was used [19] Thus, we followed these six steps for our review: (1) identifying and stating the research questions; (2) identifying relevant studies; (3) study selection; (4) data collection; (5) data summary, and synthesis of results and (6) consultation. The research questions included: (1) what are the characteristics of the studies that have examined the socio-cultural determinants of adolescent pregnancy in Ghana? and (2) what are the socio-cultural factors associated with adolescent pregnancy in Ghana?

Medical Subject Headings (MeSH) terms was used to for the search in PubMed and refined for search in other databases. Table 1 presents keywords that were moved to MeSH. A planned search strategy in PubMed is presented in Table 2. It was adapted to fit other databases (i.e. Science direct, CENTRAL, and JSTOR). Articles returned by the search were scrutinized by authors and Mendeley software was used for removing duplicates. The reference lists of all eligible articles were also scrutinized for potentially relevant articles. To include grey literature, Google Scholar and Google search were used to search for relevant materials. The last search was done on April 28, 2022. References check and institutional repository search also revealed some records. Full articles that met the eligibility criteria were finally saved in Mendeley for data charting. Two authors, MA and PA independently extracted data from articles for analysis. Details such as authors, the region where the study was conducted, the objective of the study, study design, population, study findings, and conclusions were extracted. Discrepancies that aroused from charting by the two authors (MA and PA) were resolved during meetings by all authors. Thematic and content analyses were done. Authors familiarised themselves with the extracted data, codes were assigned to data extracted to help describe the content. Authors then searched through extracted data for patterns or themes. Themes formed were then reviewed, defined, named and results presented.

**Table 1:** Keyword and MeSH

Keywords	MeSH Terms or synonyms
**Adolescent**	Adolescence, Teens, Teen, Teenagers, Teenager, Youth, Youths, Adolescents Female, Adolescent Male.
**Pregnancy**	Pregnancy, Childbirth, Pregnancies, Pregnant, Childbirths, Birth
**Sociocultural**	Communication Barriers, Norm, Social, Cultures, Belief, Customs, Cultural Background, Poverty, Peer pressure, Broken home, Child marriage, Religion, Sexual debut, Risky sexual behaviors, Substance issue, Sex conversation at home, Poor knowledge on contraception, Lack of family support, Inappropriate recreation.
**Ghana**	Republic of Ghana, Northern, Ashanti, Western, Volta, Eastern, Upper West, Central, Upper East, Greater Accra, Savannah, North East, Bono East, Oti, Ahafo, Bono and Western North.

**Table 2:** Search Strategy in PubMed

Search (#)	Search terms
1	Adolescent*[MeSH terms] OR adolescent OR adolescents[tw] OR teen* OR youth* OR young
2	Pregnancy*[MeSH terms] OR childbirth* OR Pregnancies OR Pregnant* OR Childbirths OR Birth*
3	#1 AND #2
4	Sociocultural*[MeSH terms] OR Communication Barriers* OR Norm* OR Social* OR Cultures* OR Belief* OR Customs* OR Cultural OR Poverty* OR Peer pressure* OR Broken home* Child marriage* OR Religion* OR Sexual debut* OR Risky sexual behaviors* OR Substance use* OR Sex conversation at home* OR Poor knowledge on contraception* OR family support* OR inappropriate recreation*.
5	Ghana*[MeSH term] OR Northern OR Ashanti OR Western OR Volta OR Eastern OR Upper West OR Central OR Upper East OR Greater Accra OR Savannah OR North East OR Bono East OR Oti, Ahafo, Bono OR Western North.
6	#4 AND #5 Limits: 01/01/2008 to 28/10/2021

## Eligibility criteria for considering studies for the review

### Inclusion criteria

1. Study design and participants: observational studies such as cross-sectional, case-control and cohort studies, mixed-method studies, controlled trials, qualitative studies on the socio-cultural determinants of adolescent pregnancy in Ghana.
2. Grey literature such as dissertation and thesis.
3. Study setting: Health facility or community-based studies conducted in rural or urban areas within Ghana.
4. Time period: Studies published or completed (in the case of grey literature) between January 1, 2008, and February 28, 2022, in the selected databases.
5. Language: English
6. In case of duplicate study results, the most comprehensive and current one is given priority.

### Exclusion criteria

1. Literature reviews.
2. Studies lacking the socio-cultural causes of adolescent pregnancy.
3. Studies conducted outside Ghana.
4. Conference abstracts, editorials, commentaries and letters.
5. Studies without the primary data.

### Appraisal of studies and data extraction

Briggs’s appraisal technique developed by Joanna Briggs Institute in 2011 was used to appraise included studies. This helped to certify all included studies. This tool comprises checklists for evaluating the quality of qualitative studies and cross-sectional studies as well as case-control studies. Eligible mixed-method studies were appraised with Mixed Method Appraisal Tool (MMAT) version 2018 [20]. These tools contain information on sample representativeness, appropriateness of sample size, a good description of study settings and participants, appropriate data analysis, objectivity in outcome measure, identification of sub-population, reliability, and appropriate treatment of confounding variables. A quality appraisal is done for each paper and scored low, moderate, and high based on the total number of “Yes”.

## Results

**Fig. 1:**
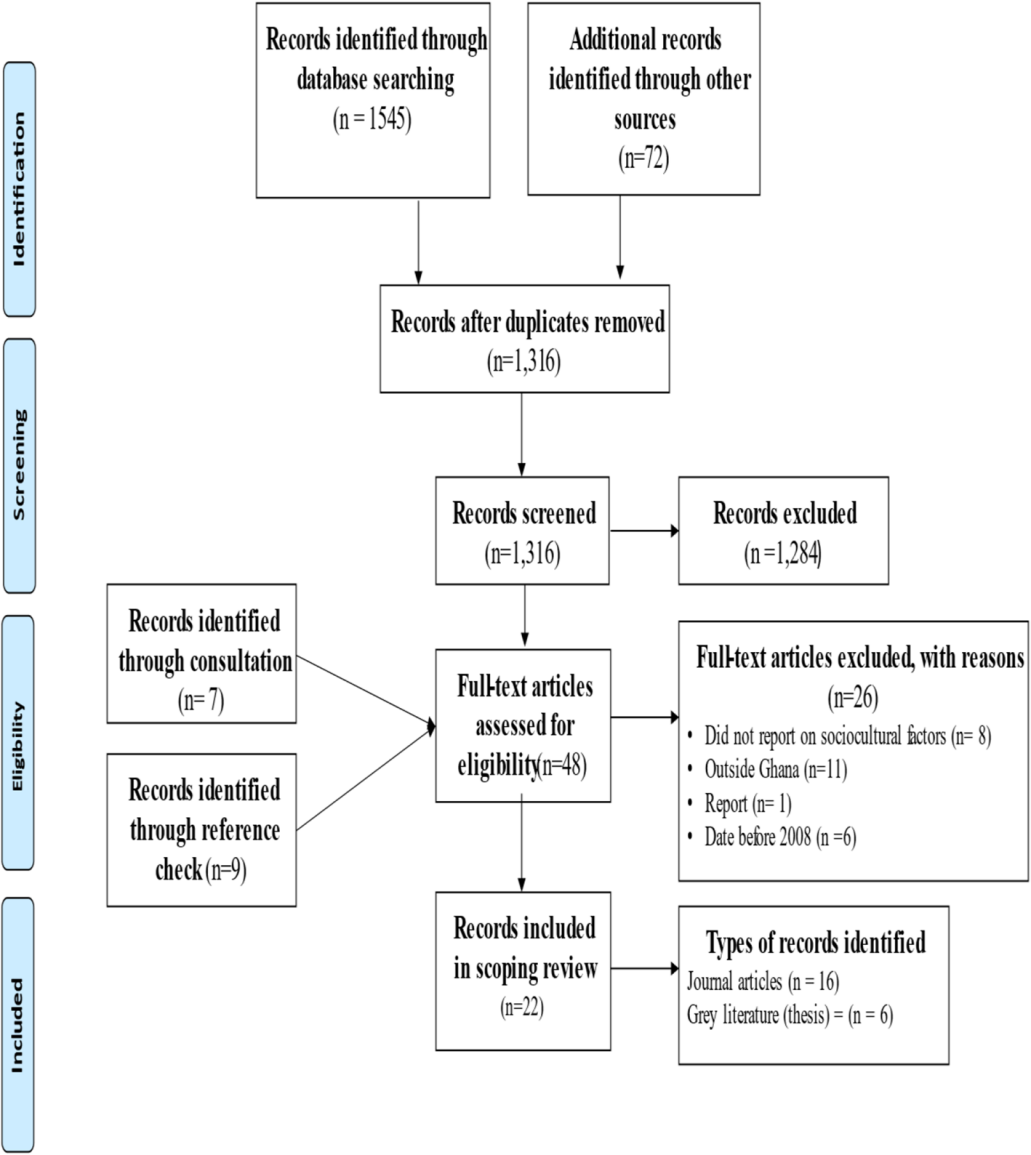
PRISMA Flow Diagram

A total of 1545 records were retrieved from the four main database (PubMed = 361, CENTRAL = 744, Science direct = 178 and Journal Storage (JSTOR) = 262). Also, 72 records were retrieved from other sources such as google, dimensions, university repositories and google scholar. In all, 1617 records were retrieved and screened for duplicates. A total of 301 duplicates were removed from the records. A detailed search result is presented in fig. 1.

## Findings

### Characteristics of the included studies

Out of the 22 studies included in this review, five were qualitative studies, eight cross-sectional studies, four case-control studies, and five mixed-method studies. We critically appraised all the included 22 studies and found only four as high, 13 as moderate and five as low quality. This means that approximately 18% of the included studies are high in quality. This also means that only a few high-quality observational studies have explored the socio-cultural determinants of adolescent pregnancy in Ghana. Moreover, only one of the included studies adjusted for confounding variables (Ahorlu et al., 2015). Details of the quality appraisal results are presented in Additional file 1 Appendix 1 to 4.

In addition, the included studies recruited 4593 adolescents, 21 teachers, 21 parents, 12 health workers and six traditional birth attendants. Most of the studies (13) recruited adolescents aged 14 years and above whereas six focused on adolescents between 10 to 19 years. However, studies that sampled adolescents aged 10 to 19 years did not do sub-group analysis to know whether socio-cultural determinants of adolescent pregnancy differ between young and old adolescents. Two studies [21, 22] did not define the adolescent age group they used in their study. Also, Alhassan [23] recruited adolescents but did not state the sample size. All 22 studies came from 10 regions out of the total 16 regions. The represented regions are Greater Accra (5), Central (5), Upper East (4), Volta (2), Ashanti (1), Bono (1), Eastern (1), Savannah (1), North East (1) and Oti Region (1). Details of study characteristics are presented in Table 3.

**Table 3:** Extracted data

Author	Purpose of the study	Design	Study Population	Sample size	Main findings	Conclusion	Appraisal score
[7]	To examine the socio-cultural factors associated with pregnancy among adolescent girls	Case-control	Adolescents (15–19)	400	Religious support for sex before marriage, strictness level of rules and regulations in the family, peer influence, are causes of teenage pregnancy.	Adolescent girls in the KEEA Municipality are likely to experience continuous exposure to the risk of pregnancy with the existence of negative socio-cultural norms.	Moderate
[10]	To investigate the factors associated with adolescent pregnancy	Case control	Adolescent (15 to 19).	245	Adolescents in rural areas and out of school, are more likely to get pregnant.	The study found three major risk factors: place of residence, occupation and economic status, as mainly associated with adolescent pregnancy.	Moderate
[40]	To examine the competencies of adolescent girls to either proactively prevent teenage pregnancy	Cross-sectional survey	Adolescents (15–19).	820	Access to cultural and social capital prevents adolescent pregnancy. Thus, access to sexual education from parents prevents adolescent pregnancy.	Focusing on developing the competencies of girls to access social, economic and cultural capital may be an effective way of tackling the threat of teenage pregnancy than focusing only on their vulnerability and associated risks.	High
[21]	To investigate the perceptions of teenagers and parents toward teenage pregnancy and teenage motherhood in Korle-Gonno	Qualitative		40	Lack of reproductive health education, lack of parental control, family and peer pressure, poverty, love for material goods, misinformation on contraception are the common causes of teenage pregnancy.	Unstable marriages, unemployment, lack of sexual reproductive health education, low –income and peer pressure are major factors that cause the high rate of teenage pregnancy in the Korle-Gonno Community.	Moderate
[22]	To assess the effect of teenage pregnancy on achieving universal basic education in Ghana	Mixed method	Teenagers	80	Lack of sex education, poor parenting, poverty, curiosity, dysfunctional family, the decline in cultural practices such as puberty rites and virginity inspection, lack of knowledge on contraception, and sexual abuse are causes of teenage pregnancy.	Teenagers principally depend on sex education from their peers and teenage and teenage pregnancy negatively impedes the goal of achieving universal basic education in Ghana.	Low
[23]	To examine the socio-economic causes of early pregnancy, psychological causes as well as its implications in Talensi.	Mixed-method	Adolescents		Family neglect, deaths of parents, lack of sex education, low and contraceptive usage, and peer pressure are the causes of teenage pregnancy.	There are many causal factors to teenage pregnancy such as funerals, mobile phones usage, peer pressure influence, lack of sex education, low usage and inadequate knowledge of contraceptives, family neglect, poverty, ignorance, sexual desire and death of parents.	Moderate
[32]	To explore how sex education could mitigate teenage pregnancy.	Mixed method	Adolescent (13–19) Teachers and head teachers.	139	Poor parenting, poverty, peer influence, concealing sex education and sex knowledge are the major causes of teenage pregnancy.	There is the need for parents and schools to empower the youth through sex education to equip them with knowledge to overcome the potentially corrupt information through the social media and friends.	Moderate
[34]	To understand the perceived decision-making preferences and determinants of early adolescent pregnancy	Qualitative (FGD)	Parents Teachers teenage (mothers and never pregnant 15–19).	56	Lack of parent-daughter communication and taboo in discussing sex related issues at home are the two main causes of teenage pregnancy.	Inadequate and inappropriate communication practices around sexuality issues, as well as weak financial autonomy is the major predictor of early adolescent pregnancy in this community.	High
[24]	To assess adolescents’ knowledge, perception and attitudes of sexual and reproductive health (SRH).	Cross-sectional survey	Adolescent (15–19).	386	Poor educational background, living non-relatives, risky sexual behaviors, perceived support from parents, poor knowledge of sexuality are risk factors for teenage pregnancy.	The findings from the study showed that the teenagers from La did not have much knowledge concerning sexual and reproductive health.	Moderate
[31]	To examine the causes, effects, and prevention of teenage pregnancy among students in Senior High Schools	Cross-sectional survey	Adolescent (15–19)	300	Per influence, lack of sexual education and poverty are the main causes of teenage pregnancy.	Parents should give their children appropriate sex education and sanction them when necessary.	Moderate
[41]	To examine factors associated with teenage pregnancy in Bolgatanga.	Qualitative	Adolescent (14–19).	20	Sexuality remains taboo, sex education in school focus on abstinence messages and the perception that apart from condom all other forms of contraception pose infertility threats.	The need for more open communication on matters of sexuality with young people and the provision of a more comprehensive sexuality education in school to address teenage pregnancies in Ghana.	High
[35]	To identify the factors associated with teenage pregnancy in the Akatsi South District.	Case-control	Adolescents (13–19).	440	Are adolescent marriage is associated with teenage pregnancy.	The study indicated that teenage pregnancy was associated with early adolescent marriage in Akatsi south district and that it was also very common among those with high socioeconomic status.	Moderate
[39]	To examine the knowledge of teenagers in teenage pregnancy and the factors that lead to teenage pregnancy	Cross-sectional	Adolescents (15–20).	20	Lack of sex education, prostitution, early sexual debut are causes of teenage pregnancy.	Teenage pregnancy can lead to several unwanted implications in the life or future of teenage girls, especially in developing countries.	Low
[27]	To examine factors associated with involvement in teenage pregnancy	Cross-sectional	Adolescents (15–19).	481	Early sexual debut and being out of school are significantly associated with teenage pregnancy.	Keeping adolescents enrolled in school might reduce their risk of involvement in pregnancy in the Ejisu- Juabeng.	Moderate
[38]	To understand why students make decisions that lead them into high-risk behavior.	Qualitative	Adolescents (15–19)	96	Peer influence, seeking financial support to stay in school, sex exchange for material things, fewer regards for STIs, and non-use of contraception are the main causes of pregnancy.	Reducing the cost of schooling for girls would reduce their need to enter into high-risk relationships, more work needs to be conducted to empower girls to negotiate their sexual rights to ensure a transition to safer sexual behavior.	Low
[26]	To assess the factors contributing to teenage pregnancy	Cross-sectional	Adolescent (10–19).	223	Married, out of school, Social media, peer influence, young age, the living situation of parents show a strong association with teenage pregnancy.	Teenage pregnancy is a public health threat in the municipality.	Low
[28]	To identify factors contributing to the rising trend in teenage pregnancy and the resultant effects.	Cross-sectional	Adolescents (10–19)	60	Poverty, no or less sexual and reproductive communication with parents, low level of education, no sexual and reproductive health knowledge, and unsafe sexual behaviors.	The following factors may account for the rising trend in teenage pregnancy in this area: family-related problems, sociocultural factors, low education level, and the lack of adequate knowledge on reproductive health.	Moderate
[29]	To explore factors contributing to repeat pregnancy among teenage mothers with repeat pregnancy.	Qualitative.	Teenagers (15–19) and health workers	41	Low level of education, out-of-school, early marriage, poverty, peer pressure, sexual coercion is the causes of pregnancy. Religious, cultural and stigma prevent teenagers from using contraceptives.	Many teenage mothers are at risk of repeat pregnancy.	High
[30]	To investigate the causes and consequences of teenage pregnancy in Assin-South District	Mixed method	Teenagers (15–19), health workers, TBAs.	102	Poor knowledge on contraceptives, peer pressure, low level of education, poverty and early marriage are causes of teenage pregnancy.	It is therefore recommended that Assin South District Health Directorate should provide much education on the use of contraceptives to teenagers in the district.	Moderate
[42]	To assess the linkages between knowledge, attitudes, and use of contraceptives and adolescent pregnancy in Komenda-Edina-Eguafo-Abrem.	Case-control	Adolescent (15–19).	196	Pregnant adolescents Embarrassing process in acquiring contraceptives, the use of traditional contraceptives, contraceptives are for married people, it feels bad to receive contraceptive information from parents and relatives and less likely to use modern contraceptives.	The findings indicate that female adolescents’ use of traditional contraceptives is associated with the risk of pregnancy in KEEA Municipality within the Central Region of Ghana.	Moderate
[33]	To investigate the major predictors of teenage pregnancy in the West Mamprusi.	Mixed-method	Adolescents (12–19 Opinion leader	Adolescents =196	Peer influence, poverty, lack of parental control, inadequate sex education and social media are the main predictors of teenage pregnancy.		Low
[25]	To identify the social determinants of non-marital adolescent pregnancy	Cross-sectional	Adolescents (10–19)	242	Low education, peer influence, Poverty and poor parent-adolescent relationship are causes of teenage pregnancy.	Non-marital adolescent pregnancy is relatively high in the Nkwanta South district and is significantly associated with no or low levels of education, poor parental relationship and poor peer relationship.	Moderate

### Socio-cultural determinants of adolescent pregnancy in Ghana

#### Low education level and lack of social amenities

Studies reported that adolescents with poor educational backgrounds or adolescents who are out of school are more likely to get pregnant [10, 24–30]. Also, adolescents in rural areas where there are scarce or no social amenities are likely to get pregnant [10].

#### Poverty and dysfunctional family

Poverty is implicated as influencing adolescent pregnancy in Ghana [21, 22, 25, 28–32]. Furthermore, issues with the adolescent immediate family or dysfunctional family may be risk factors for adolescent pregnancy. For example, poor parenting [22, 25, 32, 33], family pressure for adolescent (girls) to marry [21], parent concealing sexual education and knowledge [32], lack of parent-daughter sex communication [28, 34], strict rules and regulations in a family [7], perceived inadequate parental support [24], and parent neglect [23] are linked to adolescent pregnancy.

#### Abuse of child’s rights

Some studies reported sexual abuse of adolescent girls as a precursor of adolescent pregnancy. For instance, child marriage is a predictor of adolescent pregnancy in Ghana [26, 29, 30, 35]. Perhaps, child marriage either forced or consensual is a risk factor for adolescent pregnancy [36, 37]. Moreover, coerced sex or sexual abuse has also been reported to be a risk factor for adolescent pregnancy [22, 29].

#### Peer pressure and media influence

Aside from sexually related abuse, studies reported that peer pressure [7, 21, 23, 25, 26, 29–33, 38], social media influence [26, 33], and curiosity [22] predispose adolescents to threat of pregnancy.

#### Risky behaviours and non-use of contraception

Adolescents who engage in risky sexual behaviours such as transactional sex [24, 28, 38, 39] and early sexual debut [27, 39] are risk factors for adolescent pregnancy. Studies have also reported a lack of contraceptive usage and related issues as antecedents of adolescent pregnancy in Ghana. For instance, adolescents who have poor knowledge of sexuality or reproductive health issues [21–24, 28, 31, 33, 38–41], misconception and non-use of contraceptives [21–23, 30, 38, 41, 42], and preference for traditional contraceptives [42] are all vulnerable to adolescent pregnancy. Also, it was reported that adolescents with embarrassing experiences in the process of acquiring contraceptives are more vulnerable to pregnancy [42].

#### Cultural issues

In communities or societies where discussion of sex is treated as a taboo, adolescents are likely to deny access to essential knowledge and information regarding sexuality which may predispose them to pregnancy [34]. Finally, a study reported that a decline in cultural norms like puberty rites and virginity inspection has made more adolescents vulnerable to pregnancy [22]. Table 4 presents details on the socio-cultural determinants of adolescent pregnancy.

**Table 4:** Socio-cultural Determinants of Adolescent Pregnancy

Socio-cultural Factors	Studies
School drop-out and poor education background	[10, 24–30]
Living in rural areas	[10]
Poverty	[21, 22, 25, 28–32]
Poor parenting	[22, 25, 32, 33]
Family neglect	[23]
Family pressure	[21]
Concealing sex education and knowledge	[32]
Strick rules and regulations in family.	[7]
Lack of parent-daughter sex communication	[28, 34]
Peer influence	[7, 21, 23, 25, 26, 29–33, 38]
Curiosity	[22]
Social media	[26, 33]
Risky sexual behaviors/ transactional sex,	[24, 28, 38, 39]
Early sexual debut	[27, 39]
Perceived parent support	[24]
Poor knowledge or education on SRH	[21–24, 28, 31, 33, 38–41]
Non-usage and Misconception about contraceptives	[21–23, 30, 38, 41, 42]
Use of traditional contraceptives	[42]
Embarrassing process in acquiring contraceptives	[42]
Child-marriage	[26, 29, 30, 35]
Sexual coercion	[22, 29]
Religious support for sex before marriage	[7]
Taboo in discussing sex-related issues	[34]
A decline in puberty rites and virginity inspection	[22]

## Discussion

This scoping review found that poverty, peer influence, low level of education, dysfunctional family, lack of communication between parents and their daughters, lack of sexual and reproductive health education, child marriage, coerced sex, misconception and non-usage of contraceptives, and decline in cultural values such as puberty rites and virginity inspection are the key socio-cultural determinants of adolescent pregnancy in Ghana. In addition, there is lack of quality studies that adjust for confounding variables. Also, the decline of cultural values as determinants of adolescent pregnancy in Ghana needs further research attention. Perhaps, improving the quality of the studies might may help establish direct and combine effects of determinants of adolescent pregnancy in Ghana.

An adolescent with low education or out of school is likely not to receive sexual education. For instance, in Ghana, sex education is predominantly taught in school because it is perceived that most adolescents are in school. This approach ignores adolescents in vulnerable situations or hard-to-reach adolescents such as those on the streets, displaced, and out of school. Also, adolescents from rural areas just like the hard-to-reach adolescents mostly lack basic amenities, have difficulty accessing healthcare, sexual and reproductive health information, and are likely to drop out of school [16].

In Ghana, child marriage may be explained by cultural norms, the value placed on childbearing, betrothal, and religious traditions beliefs. A study has shown that girls who never went to school, or drop out of school and girls from low-income families are likely to marry before the age of 18 years [43]. Unfortunately, adolescents in child marriage are less involved in decisions that have an impact on their reproductive health and are less likely to use contraceptives to delay pregnancy [43]. In addition, adolescents in marriage are also likely to experience sexual abuse since they have less power to negotiate for safer sex [44]. Perhaps, coerced sex either in marriage or outside marriage exposes these young girls to pregnancy, trauma, and sexually transmitted infections, including HIV/AIDS [44, 45].

Furthermore, adolescents from poverty backgrounds might have low self-esteem due to their economic condition or live in conditions vulnerable to sexual abuse or violence [36]. In such cases, girls from low socio-economic backgrounds are likely to experience peer pressure and succumb to that or engage in sex with an older ‘rich’ person for economic gains [36]. Therefore, it makes sense for future studies to examine the complex interaction of several factors that affect teenage pregnancy which most included studies highlighted in this review.

Family disruption, dysfunctional family, and poor parenting are also risk factors for adolescent pregnancy not only in Ghana but in Africa and other low-and middle-income countries [36, 45]. In such families, there is poor communication between parents and children, neglect, and in most cases, adolescents are left to learn about sexuality on their own or the street [16]. Additionally, families or parents of poor socio-economic status put unnecessary pressure on children to marry or go into a sexual relationship in exchange for materials or financial gains [21, 23], the aim is to be able to provide food and clothing. Adolescents from dysfunctional families may rely on peers for advice or information on issues of sexuality which increases peer influence. Moreover, recent studies have reported that peer pressure and disinformation or inaccurate information from friends put adolescents at risk of sexual activities and pregnancy [36, 37, 43].

Adolescent pregnancy is also associated with a lack of or inconsistent use of contraceptives. For instance, adolescent contraceptive usage decreased from 22.1% in 2003 to 20.4% in 2014 in Ghana [46]. Moreover, adolescents in Ghana have unmet needs for contraception [5]. Unmet contraception needs are caused by limited access to contraceptives among adolescents, especially among hard-to-reach adolescents such as those living in rural areas, displaced communities, streets, refugee camps, and married adolescents [6]. Furthermore, there are misconceptions such as fear of side effects, cultural and religious restrictions, unavailability of family planning and poor-quality services that cause unmet needs for contraception among adolescents [47]. In addition, some women believe contraceptive use is linked to infertility [41]. Perhaps, adolescent girls may want to avoid pregnancy, but they may be unable to do so due to misconceptions and gaps in knowledge.

### Practical implications for policy and interventions

Determinants of adolescent pregnancy are complex and require a multidisciplinary approach to reducing such public health challenge. National efforts need to focus on creating a supportive legal and social climate as well as an enabling environment for positive adolescent development. This nurturing environment needs to ensure that adolescents have a voice, choice, and control over their bodies and can develop the capabilities required for a healthy, productive, and satisfying life.

Some adolescents become sexually active before age 14 or younger and also initiate sex early (some before age 12). This means that adolescents will need an integrated package of services which includes sexual and reproductive health services as they become sexually active. In addition, these services can include access to contraceptives such as long-acting reversible and emergency contraceptives. In addition, adolescent girls require access to safe abortion as well as management and care for unsafe abortion (i.e comprehensive abortion care). However, abortion is highly stigmatized in Ghana despite the relative abortion laws [48, 49]. Thus, abortion laws in Ghana are liberal and give rooms for abortions to be carried out for persons to protect their physical and mental health, especially adolescents. Perhaps, stigmatization of abortion, public and medical professionals’ poor knowledge of the legal status of abortion, the misconceptions surrounding the safety of legal abortion and difficult, and inadequate access to abortion services may be some of the reasons for the high prevalence of unsafe abortion among adolescents in Ghana [49]. Hence, there is a need for continuous expansion of access to legal, safe, and post-abortion care services for adolescents.

Furthermore, adolescent health service interventions require the availability of superior care, and competent and friendly healthcare workers who have been specially trained to accommodate and deal with adolescents. Reorienting health services to provide adolescent-friendly services is critical in assisting adolescents in making good reproductive health choices and improving their health. To help improve adolescent health and prevent adolescent pregnancy, the health system in Ghana must be responsive to vulnerable adolescents such as those in displaced settlements, homeless, and refugee camps and should be reached with sexual and reproductive health services and packages. Hence, policies should not ignore homeless adolescents, and safe housing programmes are needed for such groups.

Finally, quality education prepares the adolescent to avoid reproductive health risk behaviours and rather seek healthcare and services when necessary. Adolescents, particularly adolescent girls, need to pursue their education in a safe and conducive environment. Adolescent girls may require additional assistance to remain in school. Essential skills and resources such as financial literacy, life skills, safe spaces, social networks, and vocational skills and training should be tailored to the needs of adolescents. Adolescent girls should be protected and equipped with knowledge and skills in areas where child marriage is prevalent, and be encouraged and allowed to participate in decision-making as well.

### Limitations and recommendations for future studies

Most included studies were cross-sectional or descriptive studies. These studies have limitations since causality claims cannot be made. Furthermore, descriptive studies in this review did not identify confounding variables and adjust for them, which might affect the relationship between socio-cultural determinants of adolescent pregnancies. Future studies should consider longitudinal studies or high-quality observation studies that adjust for confounding variables and test moderation and mediation variables.

## Conclusion

This scoping review provides a clear and comprehensive understanding of socio-cultural risk factors influencing adolescent pregnancy in Ghana. This review revealed a complex relationship that exists between socio-cultural factors and teenage pregnancy in Ghana. In spite of the low-quality articles included in this review, we found that adolescents with low education, school drop-outs, and others living in rural and resource-constraint environments are more likely to get pregnant. Moreover, girls from dysfunctional families and those exposed to peer pressure may become pregnant. It is observed that a dysfunctional family prevents adolescents from acquiring relevant information regarding sexuality experience and the need to avoid getting pregnant during their teen age. Factors like child marriage, coerced sex or rape, early sexual debut, and transactional sex are high risk factors for adolescent pregnancy. Future studies should consider moderating variables that can reduce the effect of socio-cultural factors on adolescent pregnancy. Also, future research needs to consider longitudinal studies that adjust for confounding variables. Interventions and policies should be designed to take into consideration the needs, context, and background of adolescents. Programmes to enhance adolescent reproductive health should also consider multilevel factors where person, family, community, institutions, national, and global issues affect such programmes.

## Supplementary Information

**Additional file 1.**

## Data Availability

All data generated or analyzed during this study are included in this published article (and its supplementary information files).

## References

[1] WHO, “Adolescent pregnancy,” *World Health Organisation (WHO)*, Jan. 31, 2020. https://www.who.int/news-room/fact-sheets/detail/adolescent-pregnancy (accessed Jul. 31, 2021).

[2] Anaba AE (2017). Assessing adolescent health care quality in Ghana’s health care facilities: a study of adolescent health corners in Tema matropolis.

[3] Darroch JE, Woog V, Bankole A, Ashford LS. Costs and benefits of meeting the contraceptive needs of adolescents. New York; May 2016. Accessed: Feb. 14, 2022. [Online]. Available: https://www.guttmacher.org/adding-it-up

[4] Neal S (2012). The authors Acta Obstetricia et Gynecologica Scandinavica C 2012 Nordic Federation of Societies of. Obstet Gynecol.

[5] GSS, GHS, and ICF International. Ghana demographic and health survey 2014. Rockville, Maryland, USA; 2015. Accessed: Feb. 14, 2022. [Online]. Available: https://dhsprogram.com/pubs/pdf/fr307/fr307.pdf

[6] UNICEF. Protecting and empowering adolescent girls in Ghana. New York; 2021. Accessed: Feb. 14, 2022. [Online]. Available: https://www.unicef.org/ghana/media/4021/file/Protecting%20and%20Empowering%20Adolescent%20girls%20in%20Ghana.pdf

[7] Ahinkorah BO (2019). Examining pregnancy related socio-cultural factors among adolescent girls in the Komenda-Edina-Eguafo-Abrem Municipality in the Central Region of Ghana: A Case-control Study. Front in Public Health.

[8] Yakubu I, Garmaroudi G, Sadeghi R, Tol A, Yekaninejad MS, Yidana A (2019). Assessing the impact of an educational intervention program on sexual abstinence based on the health belief model amongst adolescent girls in northern Ghana, a cluster randomised control trial. Reprod Health.

[9] Brahmbhatt H (2014). Prevalence and determinants of adolescent pregnancy in urban disadvantaged settings across five cities. J Adolesc Health.

[10] Asare BYA, Baafi D, Dwumfour-Asare B, Adam AR (2019). Factors associated with adolescent pregnancy in the Sunyani municipality of Ghana. Int J of Afr Nurs Sci.

[11] Krugu JK, Mevissen FEF, Prinsen A, Ruiter RAC (2016). Who’s that girl? A qualitative analysis of adolescent girls’ views on factors associated with teenage pregnancies in Bolgatanga, Ghana. Reprod Health.

[12] Abebe AM, Fitie GW, Jember DA, Reda MM, Wake GE (2020). Teenage pregnancy and its adverse obstetric and perinatal outcomes at Lemlem Karl Hospital, Tigray, Ethiopia, 2018. Biomed Res Int.

[13] Neal S, Channon AA, Chintsanya J (2018). The impact of young maternal age at birth on neonatal mortality: evidence from 45 low and middle income countries. PLoS One.

[14] Kongwattanakul K, Thamprayoch R, Kietpeerakool C, Lumbiganon P (2020). Risk of severe adverse maternal and neonatal outcomes in deliveries with repeated and primary cesarean deliveries versus vaginal deliveries: a cross-sectional study. J Pregnancy.

[15] Gueye M (2020). Neonatal complications of teenage pregnancies: prospective study about 209 cases in Senegal. Am J of Pediatr.

[16] Cook SM, Cameron ST (2020). Social issues of teenage pregnancy. Obstet, Gynaecol Reprod Med.

[17] Osaikhuwuomwan JA, Osemwenkha AP (2013). Adolescents’ perspective regarding adolescent pregnancy, sexuality and contraception. Asian Pac J of Reprod.

[18] Arksey H, O’Malley L (2005). Scoping studies: towards a methodological framework. Int J of Soc Res Methodol: Theory Pract.

[19] Tricco AC (2018). PRISMA extension for scoping reviews (PRISMA-ScR): Checklist and explanation. Ann of Intern Med.

[20] Q. N. Hong et al., “Mixed methods appraisal tool (MMAT) version 2018 User guide.” pp. 1–11, 2018. [Online]. Available: http://mixedmethodsappraisaltoolpublic.pbworks.com/

[21] J. B. Aidoo, “Community and parents percetion towards teenage pregnancy and teenage motherhood at Korle-Gonno,” 2017. [Online]. Available: http://ugspace.ug.edu.gh

[22] Adu-Gyamfi E (2014). Assessing the effect of teenage pregnancy on achieving universal basic education in Ghana: a case study of upper Denkyira West District. J Educ Pract.

[23] Alhassan E (2015). Early pregnancy of junior high school girls: causes and implications on acadmeic progression in the Talensi District of the upper east Regio of Ghana. UDS Int J of Dev (UDSIJD).

[24] Abdul-Hamid I (2018). Adolescents’ knowledge, attitudes and perceptions regarding sexual and reproductive health and teenage pregnancy in La, Greater Accra region., Dissertation.

[25] Akakpo CSS (2013). Social Determinants of Non-Marital Adolescent Pregnancy in Nkwanta South District, Masters dissertation.

[26] Bedzo JY, Manortey S (2019). Factors influencing teenage pregnancy in the lower Manya Krobo municipality in the eastern region of Ghana: a cross-sectional study. OALib.

[27] Morhe ESK, Tagbor HK, Ankobea FK, Danso KA (2012). Reproductive experiences of teenagers in the Ejisu-Juabeng district of Ghana. Int J Gynecol Obstet.

[28] Nang-Bayi J, Wie SF, Siepaal V, Kuufira P, Der EM (2021). Factors associated with rising trend in teenage pregnancy within the west Gonja municipality of the Savannah region of Ghana. Open J of Obstet Gynecol.

[29] Okine L, Dako-Gyeke M (2020). Drivers of repeat pregnancy among teenage mothers in Accra, Ghana. Child Youth Serv Rev.

[30] Owusu M (2011). Teenage pregnancy in the Assin-South District, Masters Dissertation.

[31] Amoah-Saah I (2018). Causes, effects and prevention of teenage pregnancy among students in senior high schools in the Agona west municipality in the Central region, Ghana, Masters Thesis.

[32] Donkor AK, Lariba AL (2017). The impact of sex education on teenage pregnancy in basic schools of Bawku municipal district in Ghana. J Pendidikan Biol Indones.

[33] Ziblim S-DS. So many teen mothers in my village: factors contributing to teenage pregnancy in west Mamprusi District in Ghana. iJARS Int J Humanit Soc Stud. 2017;3(6):1–11. 10.20908/ijarsijhss.v3i06.10428.

[34] Bain LE, Muftugil-Yalcin S, Amoakoh-Coleman M, Zweekhorst MBM, Becquet R, de Cock Buning T (2020). Decision-making preferences and risk factors regarding early adolescent pregnancy in Ghana: stakeholders’ and adolescents’ perspectives from a vignette-based qualitative study. Reprod Health.

[35] Kyeremeh N (2018). Factors associated with teenage pregnancy in Akatsi South District of the Volta region, Masters dissertation.

[36] Chung HW, Kim EM, Lee JE (2018). Comprehensive understanding of risk and protective factors related to adolescent pregnancy in low- and middle-income countries: a systematic review. J Adolesc.

[37] Gunawardena N, Fantaye AW, Yaya S (2019). Predictors of pregnancy among young people in sub-Saharan Africa: A systematic review and narrative synthesis. BMJ Global Health.

[38] Mac Domhnaill B, Hutchinson G, Milev A, Milev Y (2011). The social context of schoolgirl pregnancy in Ghana. Vulnerable Child Youth Stud.

[39] Adzitey SP, Adzitey F, Suuk L (2013). Teenage pregnancy in the Builsa District: a focus study in Fumbisi. JLSB J of Life Sci Biomed.

[40] Ahorlu CK, Pfeiffer C, Obrist B (2015). Socio-cultural and economic factors influencing adolescents’ resilience against the threat of teenage pregnancy: a cross-sectional survey in Accra, Ghana adolescent health. Reprod Health.

[41] Krugu JK, Mevissen F, Münkel M, Ruiter R (2017). Beyond love: a qualitative analysis of factors associated with teenage pregnancy among young women with pregnancy experience in Bolgatanga, Ghana. Cult Health Sex.

[42] Ahinkorah BO (2021). Linking female adolescents’ knowledge, attitudes and use of contraceptives to adolescent pregnancy in Ghana: a baseline data for developing sexuality education Programmes. Healthcare.

[43] Ahonsi B (2019). Child marriage in Ghana: evidence from a multi-method study. BMC Womens Health.

[44] de Groot R, Kuunyem MY, Palermo T (2018). Child marriage and associated outcomes in northern Ghana: a cross-sectional study on behalf of the Ghana LEAP 1000 evaluation team. BMC Public Health.

[45] Kassa GM, Arowojolu AO, Odukogbe AA, Yalew AW (2018). Prevalence and determinants of adolescent pregnancy in Africa: A systematic review and Meta-analysis 11 Medical and Health Sciences 1117 Public Health and Health Services. Reprod Health.

[46] Appiah F, Seidu AA, Ahinkorah BO, Baatiema L, Ameyaw EK (2020). Trends and determinants of contraceptive use among female adolescents in Ghana: analysis of 2003–2014 demographic and health surveys. SSM - Popul Health.

[47] Guure C (2019). Factors influencing unmet need for family planning among Ghanaian married/union women: a multinomial mixed effects logistic regression modelling approach. Arch of Public Health.

[48] Bain LE (2019). To keep or not to keep? Decision making in adolescent pregnancies in Jamestown, Ghana. PLoS One.

[49] Guttmacher Institute. Incidence of abortion and provision of abortion-related Services in Ghana. New York; May 2020. Accessed: Aug. 22, 2021. [Online]. Available: https://www.guttmacher.org/sites/default/files/factsheet/incidence-abortion-and-provision-abortion-related-services-ghana.pdf

