# Supplementary material for: Socio-cultural factors influencing adolescent pregnancy in Ghana: a scoping review
Source: BMC Pregnancy Childbirth. 2022 Nov 11;22:834. doi: 10.1186/s12884-022-05172-2 (PMC9652868; doi:10.1186/s12884-022-05172-2)
Supplement: Supplementary file 1 — Additional file 1. [file 12884_2022_5172_MOESM1_ESM.docx]

**Appendix 1: JBI critical appraisal checklist for eligible qualitative studies**

| **Checklist** | [34] | [41] | [21] | [38] | [29] |
| --- | --- | --- | --- | --- | --- |
| 1. Is there congruity between the stated philosophical perspective and the research methodology? | Unclear | No | No | No | Yes |
| 1. Is there congruity between the research methodology and the research question or objectives? | Yes | Yes | Yes | Yes | Yes |
| 1. Is there congruity between the research methodology and the methods used to collect data? | Yes | Yes | Yes | Yes | Yes |
| 1. Is there congruity between the research methodology and the representation and analysis of data? | Yes | Yes | Yes | Yes | Yes |
| 1. Is there congruity between the research methodology and the interpretation of results? | Yes | Yes | Yes | Yes | Yes |
| 1. Is there a statement locating the researcher culturally or theoretically? | No | No | No | No | Yes |
| 1. Is the influence of the researcher on the research, and vice- versa, addressed? | Yes | Yes | No | No | Yes |
| 1. Are participants, and their voices, adequately represented? | Yes | Yes | Yes | No | Yes |
| 1. Is the research ethical according to current criteria or, for recent studies, and is there evidence of ethical approval by an appropriate body? | Yes | Yes | Unclear | Unclear | Yes |
| 1. Do the conclusions drawn in the research report flow from the analysis, or interpretation, of the data? | Yes | Yes | Yes | Yes | Yes |
| 1. Included/Excluded | Included | Included | Included | Included | Included |
| 1. Overall appraisal   Low = 5 yes or less  Moderate= 6 to 7 yes  High = 8 yes or more | High | High | Moderate | Low | High |

**Appendix 2: JBI critical appraisal checklist for eligible cross-sectional studies**

| **Checklist** | [24] | [40] | [31] | [39] | [27] | [26]. | [28] | [25] |
| --- | --- | --- | --- | --- | --- | --- | --- | --- |
| 1. Were the criteria for inclusion in the sample clearly defined? | Yes | Yes | Yes | Yes | Yes | No | Yes | Yes |
| 1. Were the study subjects and the setting described in detail? | Yes | Yes | No | Yes | No | Yes | Yes | Yes |
| 1. Was the exposure measured in a valid and reliable way? | Unclear | Yes | Yes | Unclear | Unclear | No | Yes | Yes |
| 1. Were objective, standard criteria used for measurement of the condition? | Yes | Yes | Yes | Unclear | Yes | Unclear | Yes | Yes |
| 1. Were confounding factors identified? | No | Yes | No | No | No | No | No | No |
| 1. Were strategies to deal with confounding factors stated? | No | Yes | No | No | No | No | No | No |
| 1. Were the outcomes measured in a valid and reliable way? | Yes | Yes | Yes | No | Yes | Unclear | Yes | Yes |
| 1. Was appropriate statistical analysis used? | Yes | Yes | Yes | Yes | Yes | Yes | Yes | Yes |
| 1. Included/Excluded | Included | Included | Included | Included | Included | Included | Included | Included |
| 1. Overall appraisal   Low = 4 yes or less  Medium = 5 to 6 yes  High = 7 yes or more | Moderate | High | Moderate | Low | Moderate | Low | Moderate | Moderate |

**Appendix 3: JBI critical appraisal checklist for eligible case-control studies**

| **Checklist** | [10] | [35] | [7] | [42] |
| --- | --- | --- | --- | --- |
| 1. Were the groups comparable other than the presence of disease in cases or the absence of disease in controls? | Yes | Yes | Yes | Yes |
| 1. Were cases and controls matched appropriately? | Yes | No | Yes | Yes |
| 1. Were the same criteria used for identification of cases and controls? | Yes | Yes | Yes | Yes |
| 1. Was exposure measured in a standard, valid and reliable way? | Yes | Yes | Yes | Yes |
| 1. Was exposure measured in the same way for cases and controls? | Yes | Yes | Yes | Yes |
| 1. Were confounding factors identified? | No | No | No | No |
| 1. Were strategies to deal with confounding factors stated? | No | No | No | No |
| 1. Were outcomes assessed in a standard, valid and reliable way for cases and controls? | Yes | Yes | Yes | Yes |
| 1. Was the exposure period of interest long enough to be meaningful? | No | No | No | No |
| 1. Was appropriate statistical analysis used? | Yes | Yes | Yes | Yes |
| 1. Included/Excluded | Included | Included | Included | Included |
| 1. Overall appraisal   Low = 5 yes or less  Medium = 6 to 7 yes  High = 8 yes or more | Moderate | Moderate | Moderate | Moderate |

**Appendix 4: JBI critical appraisal checklist for eligible mixed-methods studies**

| **Checklist** | [32] | [23] | [30] | [22] | [33] |
| --- | --- | --- | --- | --- | --- |
| 1. Are there clear research questions? | Yes | Yes | Yes | Yes | Yes |
| 1. Do the collected data allow to address the research questions? | Yes | Yes | Yes | Yes | Yes |
| 1. Is there an adequate rationale for using a mixed methods design to address the research question? | No | Yes | Yes | Yes | Yes |
| 1. Are the different components of the study effectively integrated to answer the research question? | Yes | Yes | Yes | No | No |
| 1. Are the outputs of the integration of qualitative and quantitative components adequately interpreted? | Yes | Unclear | Yes | No | No |
| 1. Are divergences and inconsistencies between quantitative and qualitative results adequately addressed? | No | Unclear | No | No | No |
| 1. Do the different components of the study adhere to the quality criteria of each tradition of the methods involved? | N0 | No | Unclear | No | No |
| 1. Included/Excluded | Included | Included | Included | Included | Included |
| 1. Overall appraisal   Low = 3 yes or less  Medium = 4 to 5 yes  High = 6 yes or more | Moderate | Moderate | Moderate | Low | Low |
